# Indoor Environmental Quality in Dwellings and Lifestyle Behaviors during the COVID-19 Pandemic: Russian Perspective

**DOI:** 10.3390/ijerph18115975

**Published:** 2021-06-02

**Authors:** Vladimir Reshetnikov, Oleg Mitrokhin, Elena Belova, Victor Mikhailovsky, Maria Mikerova, Ali Alsaegh, Irina Yakushina, Valery Royuk

**Affiliations:** 1 N.A. Semashko Department of Public Health and Healthcare, I.M. Sechenov First Moscow State Medical University, Bld. 2, 2 Bolshaya Pirogovskaya Str., Moscow 119435, Russia; resh1960@mail.ru (V.R.); buzzdoc@yandex.ru (V.M.); masha-med@mail.ru (M.M.); yakushina_i_i@staff.sechenov.ru (I.Y.); vvroyuk@mail.ru (V.R.); 2 Department of General Hygiene, I.M. Sechenov First Moscow State Medical University, Bld. 2, 2 Bolshaya Pirogovskaya Str., Moscow 119435, Russia; mov1163@yandex.ru (O.M.); ms.ekochina@mail.ru (E.B.)

**Keywords:** COVID-19, Russia, self-isolation, indoor environment, built environment, building codes, ventilation, sewage, disinfection, lifestyle

## Abstract

The novel coronavirus (COVID-19) outbreak is a public health emergency of international concern, and as a response, public health authorities started enforcing preventive measures like self-isolation and social distancing. The enforcement of isolation has consequences that may affect the lifestyle-related behavior of the general population. Quarantine encompasses a range of strategies that can be used to detain, isolate, or conditionally release individuals or populations infected or exposed to contagious diseases and should be tailored to circumstances. Interestingly, medical students may represent an example of how the COVID-19 pandemic can form new habits and change lifestyle behaviors. We conducted a web-based survey to assess changes in lifestyle-related behavior of self-isolated medical students during the COVID-19 pandemic. Then we analyzed the sanitary-hygienic regulations of the Russian Federation to determine the requirements for healthy buildings. Results showed that during the pandemic, the enforcement of isolation affects medical students’ lifestyle-related behavior and accompanies an increase in non-communicable diseases (NCDs). Indoor environmental quality (IEQ) and healthy buildings are cutting-edge factors in preventing COVID-19 and NCDs. The Russian sanitary-hygienic regulations support improving this factor with suitable requirements for ventilation, sewage, waste management, and disinfection. Herein, assessing isolation is possible through the hygienic self-isolation index.

## 1. Introduction

According to the World Health Organization (WHO, 2008), lifestyles are essential determinants of health, and the latest studies confirmed this. Lifestyles can be affected by many factors, which include public health measures such as quarantine and isolation. Those measures can affect the social ties and the lifestyle habits of the general population. Nonetheless, many scientific reports proved the effectiveness of these measures in containing outbreaks [1,2,3,4,5,6,7,8].

Isolation refers to the separation and restricted movement of ill persons who have a contagious disease and suspected infected persons to prevent transmission to others. It typically occurs in a hospital setting but can be done at home or in a special facility. Usually, individuals are isolated, but the practice may be applied in larger groups. Quarantine encompasses a range of strategies that can be used to detain, isolate, or conditionally release individuals or populations infected or exposed to contagious diseases and should be tailored to circumstances [9,10].

Many studies strongly recognize the indoor environment as a major health determinant. People spend up to 90% of their lifetime indoors. The occurrence and re-occurrence of pathologies related to the quality of the built environment and exacerbated by the severe current socio-economic crisis uphold once more the utmost importance of the domestic environment as a principal living space [9,11].

Self-isolation and quarantine are effective sanitary-hygienic measures that aim to minimize infectious risk in public places. For people in dwellings during the enforcement of self-isolation, there are three preventive approaches: (1) telecommuting (remote working) and distance education, (2) partial isolation, and (3) full quarantine [12,13].

Moscow and the Moscow Region Podmoskovye are the most affected regions in the Russian Federation since the novel coronavirus (COVID-19) pandemic began. On 26 March 2020 (the time of enforcing quarantine), 136 COVID-19 cases were registered in Moscow and Podmoskovye; this number represents 74.7% of the total cases registered in Russia (182). On 11 May 2020 (during the period of our survey and the second COVID-19 wave in Russia), the highest record of COVID-19 cases was registered; in Moscow and Podmoskovye, the total number of registered cases was 52.9% (6162) of the registered cases in Russia (11,656). As a response, they introduced a set of measures that mainly focused on limiting social contacts to prevent the spread of COVID-19 [14].

Evidence-based efforts are essential, and many methodologies and measures are being actively studied and developed to prevent the spread of COVID-19 and other infectious diseases. One of them is self-isolation, which represents a public health measure that aims to minimize contact with people from outside the home environment [15].

From a hygienic viewpoint, housing-related factors such as ventilation, sewage, waste management, and disinfection affect human health. Failure to comply with the building code can harm health, and supporting a healthy indoor environment during isolation is essential in preventing the spread of coronavirus infection; the time spent at home reaches almost 100%. Law enforcement is a key part of public health protection in an indoor environment, and a general re-thinking of housing policies and regulations is mostly needed. Therefore, self-isolation may lead to the formation of factors that harm the human body, such as physical inactivity, hypoxia, nutritional factors, changes in work and rest, and psycho-emotional stress [11,16,17,18,19].

During isolation, carrying out disinfection to sterilizing airflow in air-conditioning and ventilation systems is an essential measure for maintaining the spread of coronavirus infection, especially in dwellings, where chances of being exposed to environmental factors that can affect human health are higher [20].

Isolation may limit physical activity and, as a result, lead to an increase in body weight, an imbalanced diet, and a greater susceptibility to bad habits. The enforcement of isolation can significantly influence the social ties and the lifestyle habits of the population. It can also change the usual daily routine and limit communication; many organizations switched to remote working and studying [21,22,23].

The lifestyle of isolated students can lead to a decrease in social contacts, a formation of new habits (wearing masks, sanitizing, social distancing), an increase in anxiety and stress, low sleep quality, more frequent use of gadgets, and physical inactivity [24,25,26,27,28].

Interestingly, medical students have a unique lifestyle; obtaining a medical degree is a long pathway, and the academic workload in medical school is very high. Accordingly, it is imperative to consider those specificities while protecting the health of medical students. Moreover, the increase in mental and physical workload and the decrease in students’ physical activity highlights the importance of developing approaches for promoting a healthy educational environment [29,30,31,32,33].

This article aims to identify lifestyle behaviors during the enforcement of isolation and analyze the Russian regulations that standardize the indoor environment in dwellings which play a crucial role in those changes. Russian medical students and their unique lifestyles represented a target group to our study.

## 2. Materials and Methods

We conducted a web-based survey using an online survey platform Anketolog, from 14 May to 26 May 2020, to assess the changes in lifestyle-related behavior of self-isolated medical students at Sechenov University, Moscow, Russia, during the novel coronavirus (COVID-19) pandemic. While conducting this study, we considered that the university canceled all face-to-face classes because of applied coronavirus (COVID-19) preventive measures. We stratified the involved students according to the university academic year group and study program. The university has around 18,000 students. Statistically, there is a need for a sample of 376 enrolled individuals to investigate the selected variables in the population (students), assuming that there is a 95% confidence level and a 5% margin of error. The total number of involved participants (respondents) is 761.

Inclusion criteria: Russian-speaking medical students at Sechenov University that were in Moscow, Russia, during the implementation of isolation orders. All included participants agreed on voluntary participation. Exclusion criteria: Not satisfying the inclusion criteria.

Areas of question included the socio-demographic background of the participants (age, gender, marital status, and residence) and the following self-isolation lifestyle-related aspects: maintaining a healthy lifestyle, physical activity, sleep patterns, daily routine, workload, diet, addiction, bad habits, mental health, and COVID-19 precautions. The study did not include any experiments involving human or biological human samples, nor any research on identifiable human data. Participants expressed their informed consent prior to filling in the questionnaire. We performed a statistical analysis using IBM SPSS Statistics 20.0 (IBM, Chicago, IL, USA) and summarized all extracted data as either words or percentages. Then we analyzed data from the sanitary-hygienic regulatory framework of the Russian Federation to determine the Russian governmental standards and recommendations that aim for achieving a healthy indoor environment.

## 3. Results

Most respondents (81.1%) are females, and 18.9% are males. The average age of the respondents is 20.2 (from 17 to 39 years). Most of them (65.8%) are single, 12.9% have common-law partners, and 3.2% are married. Among all of them, only 2.0% have children. Most of them (52.8%) live in apartments with friends and/or relatives, 21.4% live in private apartments, 9.1% live in owned houses, and 8.0% in rented houses.

### 3.1. Healthy Lifestyle

Only a third of the respondents (31.1%) are confident about maintaining a healthy lifestyle, and 15.1% registered an unhealthy lifestyle. More than half of the respondents (53.6%) answered that the high workload disturbs them from maintaining a healthy lifestyle, 48.9% admitted that they are too lazy to maintain a healthy lifestyle, 40.1% reported a lack of desire and motivation, and 20.0% a lack of financial resources for healthy food.

### 3.2. Physical Activity

Physical inactivity is one of the factors of an unhealthy lifestyle in the self-isolated population. Only 11.3% of respondents exercise every day, and 14.8% do not exercise at all. Those who engage in physical activity spend around 72.6 ± 3.0 min a day on this activity. Most of them (64.0%) are independently engaged in physical activity, 35.5% use video resources for this purpose, and 21.8% keep fit using special devices and smartphone applications. A significant part (41.8%) does not engage in physical activity due to the lack of time due to a high workload, and a third (36.0%) has no desire.

### 3.3. Sleep Patterns

More than half of the respondents (53.9%) reported a sleep duration of 6–8 h, and 27.9% reported more than 8 h. However, 3.3% of respondents reported a significant day-to-day sleep variation (from 1 to 16 h). A noteworthy part of respondents (47.7%) reported an unstable ability to get enough sleep, and 12.4% suffered from chronic sleep deprivation. Half of the respondents (50.2%) did not get the required amount of sleep due to a high workload, 44.4% preferred to surf the web, and 25.0% enjoyed hobbies or considered sleeping as a hobby.

### 3.4. Daily Routine

Only a fifth of respondents (21.9%) have a healthy daily routine. Analysis of the distribution of time per day showed that, on average, one-third of the time (31.2%) they spent on sleep, 20.5% on independent learning, 14.8% on online classes, 10.0% on household chores and hobbies, 5.5% on walking and getting fresh air, 5.0% on physical activity, and 3.0% of the day on watching programs to get information about COVID-19.

### 3.5. Study Workload

On average, respondents spend 3.5 h a day on online classes and about 5 h a day on independent preparation for those classes. The respondents that evaluated the workload as very high were 26.2%, and 37.3% evaluated it as high. During self-isolation, workload and distance education are both associated with the use of gadgets; 2.7% of respondents spend all day in front of a monitor/tablet/phone screen. On average, respondents spend more than 8 h a day in front of a monitor; most of them (94.9%) used gadgets to study, 81.9% to visit social networks, and 79.0% to communicate.

### 3.6. Diet

One-fourth of respondents (24.0%) maintain a healthy eating lifestyle, and 21.6% do not. A third of the respondents (33.6%) reported that the high workload is a disturbing factor for maintaining a healthy eating lifestyle. Statistically, there was no significant differences in the body mass index (BMI) before and after the enforcement of isolation = 21.55 kg. The BMI in females was 21.1 kg and 23.61 kg before and after the enforcement of isolation, respectively. The BMI in males was 23.5 kg and 23.61 kg before and after the enforcement, respectively.

### 3.7. Addiction and Bad Habits

Thirty percent of respondents confirmed that they have addictions, 18.8% smoke, and 42.8% consume alcoholic beverages. Furthermore, a way to relieve tension and stress is smoking for 20.8% of respondents and consuming alcohol for 38.6%. However, due to the enforcement of COVID-19 isolation measures, 18.2% of respondents said they smoked more, and 30.8% said they smoked less. Nevertheless, also due to isolation measures, 5.0% indicated that they quit smoking during the period of self-isolation. Regarding alcoholic beverage consumption: 14.1% of respondents tend to drink more, and 13.8% tend to drink less.

### 3.8. Health

Almost half of the respondents (45.7% and 45.2%) reported daily fatigue and internal stress, respectively. A third of the respondents (32.1% and 33.6%) experienced a feeling of despair and anxiety, respectively. Besides, 37.4% experienced irritability, and 37.5% experienced daily discomfort in their eyes.

### 3.9. COVID-19 Precautions

The responses showed that 7.6% of respondents do not consider any COVID-19 precautions, 72.5% wash their hands more often, 73.3% use personal protective equipment (PPE), and 70.3% avoid social contacts.

When going outside, 81.1% of respondents wear masks, and 53.4% use protective gloves. When entering the subway, shops, public places, 85.0% of respondents wear masks, 62.4% use gloves, and 48.4% wash their hands with antiseptics.

As respondents return home, 94.6% wash their hands, 59.9% wash their face, 39.7% wash their nose, 66.4% wash their hands with antiseptics, 61.9% wipe surfaces with antiseptics, and 84.8% change their shoes. Moreover, 43.6% of respondents wash their hands after visiting public places, 29.6% once every 2–3 h, and 24.2% of the respondents wash hands as frequently as before the enforcement of self-isolation. Furthermore, less than one percent of the respondents reported that they wash their hands after using gadgets.

## 4. Sanitary-Hygienic Regulatory Framework of the Russian Federation

The Russian Government has sanitary-hygienic standards for nutrition, physical activity, work, and rest. Those standards determine the requirements of physiologically optimal conditions for staying at home, microclimate indicators, physical activity, modes of work and rest, and the estimated energy requirements (EER) that determine the physiological needs for energy and nutrients of various groups of the population. Further, according to those standards, preventing the spread of coronavirus infection (COVID-19) in the living environment is an essential issue for the national public health authorities [2,34,35,36,37,38].

For optimal hygienic self-isolation, an essential issue is the efficiency of the arrangements and maintenance of the whole-house ventilation system, as well as COVID-19 disinfection of the house. Carrying out the passive ventilation of residential premises should be performed by airflow through vents, transoms, or through special openings in window sashes and ventilation ducts. Air exhaust openings should be provided in kitchens, bathrooms, toilets, and drying cabinets, and ventilation systems should exclude airflow from one apartment to another [39].

Furthermore, an important issue is the minimum ventilation rate per person. This issue depends on the living space of an apartment building: for rooms over 20 m^2^ with passive ventilation, the minimum outdoor air consumption is 30 m^3^/h, and without passive ventilation 60 m^3^/h, and in a room with a living area of less than 20 m^2^, the minimum ventilation rate is 3 m^3^/h per 1 m of living space [40,41].

Air Change Rates Per Hour (ACPH) of the bedroom, living room, and children’s rooms during a non-operating mode are 0.2 m^3^ per hour, during an operating mode 1.0 m^3^ per hour. The ACPHs of libraries and offices are 0.2 and 0.5, respectively. The ACPHs of bathrooms, showers, restrooms, and combined bathrooms are 0.5–25. Removal of air should be from kitchens, toilets, bathrooms, and if necessary, from other rooms. Moreover, while installing adjustable ventilation, it is essential to provide grilles and valves on exhaust ducts and air ducts. Indoor air, which contains harmful substances or unpleasant odors, must be directly removed in anticipating its entrance to any other rooms. Furthermore, combining ventilation ducts from kitchens, toilets, bathrooms (showers), combined lavatories, food storage rooms with ventilation ducts from rooms with gas-powered equipment, and parking lots is not allowed [42].

It is necessary to clean the heaters once every six months and to inspect and clean the filters and replace them as they become dirty once a month. Further, it is possible to calculate the surface area by multiplying the duct’s circumference (meters) by its height and depends on the duct length. Thus, it is possible to calculate the required amount of disinfectant. Moreover, wiping or sprinkling are the only methods for carrying out disinfection of the ventilation elements and the air conditioning systems. It is necessary to check the cleanliness of air intake and air ducts at least once a year. Moreover, disinfection of the ventilation and air-conditioning systems should be carried out at least once a quarter with a complete shutdown of the system [43].

## 5. Discussion

Based on the sanitary-hygienic standards of the Russian Federation, dwellings can be permanent (apartments and country houses) for families and temporary (hotels and hostels) for medical personnel [44]. However, during pandemics, they can also be classified considering two fundamental aspects. The first aspect is the registration of contact with an infected or ill person. Prevention approaches include but are not limited to reducing the risk of disease transmission [4]. The second aspect is the long-term stay of healthy people due to the implication of teleworking, movement restrictions, and curfew. Prevention approaches include but are not limited to reducing the risk of infection, enduring a balanced diet and physical activity, and staying in the fresh air to prevent non-communicable diseases [4,5,6].

The analysis of the published literature showed that scenarios for controlling the spread of COVID-19 infection in the indoor environment are the following:

1. Telecommuting (remote working) and distance education during isolation, quarantine, and lockdown [20,21,22].
2. Partial isolation: when one of the family members tests positive for COVID-19 or for a mild disease, and the other family members isolate or stay under medical supervision [21].
3. Full isolation: when all family members test positive for COVID-19 or for a mild disease and are not allowed to leave their living environment and are monitored with contact tracing mobile applications [22].

The sanitary-hygienic aspects of controlling the spread of COVID-19 in the living environment include the design of ventilation and sewerage system, cleaning, and disinfecting premises [40].

Disinfection is an effective measure for residential buildings and for the isolated population, ensuring safety in apartments. In houses, it is necessary to carry out wet cleaning at least two times a day and should include all rooms. Further, while treating surfaces, special attention should be given to surfaces that sick people and their family members touch frequently such as doorknobs, taps, tables, and chair backs.

COVID-19 infected people should use separate towels and beddings from other family members; they may be contaminated by a biological agent and bodily fluids containing pathogens. Besides, before washing, it is necessary to soak textiles in solutions of disinfectants, and generally, washing should be at a temperature of at least 60 degrees. They also must eat separately and use separate dishes, and after using them, disinfection should be carried out [25].

Further, waste should be packed in extra-strong contractor garbage bags, and volunteers should help in waste management [37].

Sewage treatment is an essential hygiene issue that should be analyzed. In Russia, the wastewater disinfection of residential buildings is carried out in city-wide treatment plants, and those plants are standardized according to parasitological and microbiological safety requirements (E. coli, Pathogenic Microorganisms, Helminth eggs, Biohelminths, and Intestinal Protozoan Cysts). The issue of wastewater disinfection during the coronavirus pandemic is not well studied. The discovery of SARS-CoV-2 encouraged several research groups around the world to study this issue. China has developed guidelines on technologies for wastewater disinfection with a coronavirus accent. The presence of SARS-CoV-2 genetic material in wastewater can be used to monitor the spread of COVID-19 [31,32,33,34,35].

Studies from three Italian universities proved that restrictive measures such as isolation and the closure of fitness facilities led to a decrease in the physical activity of the general population [45]. Many studies showed a decrease in the level of physical activity of students, especially those who usually walk (in our study, 20.0% of participants answered they do not walk at all). Setting for a long time and an increase in the time spent in front of the monitor (the results of our study showed that students spend about eight and a half hours using smartphone/computer screens) [46].

During an international emergency like COVID-19, an adequate assessment of public health measures like self-isolation is essential. Based on sanitary-hygienic criteria, the hygienic self-isolation index (HISI) can assess the risk of self-isolation. This index is convenient and beneficial for establishing a comprehensive assessment for self-isolation and determining the isolation compliance level using all the recommended physiological-hygienic standards. HISI determines that an optimal self-isolation is directly proportional to the coefficients of a person’s physical activity (D), indoor area (air cubic capacity) per isolated person (S), time spent in fresh air (T); and inversely proportional to the calorie intake (K), and the total number of domestic conflicts (C).

$HISI=\frac{D+S+T}{K+C}$; (Table 1).

Limitations of the study: after implementing an objective assessment of the sanitary-hygienic regulations of the indoor environment of dwellings during the enforcement of self-isolation, it is difficult to conclude any association between lifestyle behaviors and the indoor environment. All the results of the study are subjective.

## 6. Conclusions

- During the novel coronavirus (COVID-19) pandemic, isolation and social distance are essential measures for containing the spread of COVID-19.
- Results of the study showed that the lifestyle behaviors characteristics of medical students during the enforcement of COVID-19 preventive measures are higher academic workload, unhealthy eating habits, unhealthy daily routine, increased alcohol consumption, stress, and anxiety [48,49,50].
- Risk factors of the indoor environment during the enforcement of COVID-19 in-house preventive measures are the following: ventilation, sewage, wastes, and contaminated surfaces.
- Measures to prevent the spread of COVID-19 included carrying out ventilation, waste management, and wastewater disinfection [51,52].

## Figures and Tables

**Table 1:** The sanitary-hygienic criteria of the hygienic self-isolation index (HISI).

Criteria for Evaluation	Value
Physical activity of an isolated person (D)	The actual physical activity (the number of calories spent on each physical exercise)/exercise time. (Recommendations of the World Health Organization) [38].
Space of isolation (cubic meter of air) per isolated person (S)	The actual space (cubic capacity) of self-isolation area: 1. In a 20 m^2^ total area per person: K = 3 m^3^/h. 2. If the total area is not less than 20 m² per person: K = not less than 30 m^3^/h [39].
Time spent on outdoor activities (T)	Time spent on getting fresh air (hours).
Calorie or energy intake (K)	The caloric value of food (indicated on the product labels); physiological energy requirements for adults from 2100 to 4200 kcal/day for males and from 1800 to 3050 kcal/day for females [39,40,41,47].
Number of domestic conflicts (C) per week	

## Data Availability

The data presented in this study are available on request from the corresponding author. The data are not publicly available to protect confidentiality of the research participants.
